# A Convenient *In Vivo* Model Using Small Interfering RNA Silencing to Rapidly Assess Skeletal Gene Function

**DOI:** 10.1371/journal.pone.0167222

**Published:** 2016-11-28

**Authors:** Wen Zhang, Can Liu, Bao Hai, Guohong Du, Hong Wang, Huijie Leng, Yingsheng Xu, Chunli Song

**Affiliations:** 1 Department of Orthopaedics, Peking University Third Hospital, Beijing, China; 2 Beijing Key Laboratory of Spinal Diseases, Beijing, China; 3 Department of Neurology, Peking University Third Hospital, Beijing, China; Universite de Nantes, FRANCE

## Abstract

It is difficult to study bone in vitro because it contains various cell types that engage in cross-talk. Bone biologically links various organs, and it has thus become increasingly evident that skeletal physiology must be studied in an integrative manner in an intact animal. We developed a model using local intraosseous small interfering RNA (siRNA) injection to rapidly assess the effects of a target gene on the local skeletal environment. In this model, 160-g male Sprague-Dawley rats were treated for 1–2 weeks. The left tibia received intraosseous injection of a *parathyroid hormone 1 receptor* (*Pth1r*) or *insulin-like growth factor 1 receptor* (*Igf-1r*) siRNA transfection complex loaded in poloxamer 407 hydrogel, and the right tibia received the same volume of control siRNA. All the tibias received an intraosseous injection of recombinant human parathyroid hormone (1–34) (rhPTH (1–34)) or insulin-like growth factor-1 (IGF-1). Calcein green and alizarin red were injected 6 and 2 days before euthanasia, respectively. IGF-1R and PTH1R expression levels were detected via RT-PCR assays and immunohistochemistry. Bone mineral density (BMD), microstructure, mineral apposition rates (MARs), and strength were determined by dual-energy X-ray absorptiometry, micro-CT, histology and biomechanical tests. The RT-PCR and immunohistochemistry results revealed that IGF-1R and PTH1R expression levels were dramatically diminished in the siRNA-treated left tibias compared to the right tibias (both p<0.05). Using poloxamer 407 hydrogel as a controlled-release system prolonged the silencing effect of a single dose of siRNA; the mRNA expression levels of IGF-1R were lower at two weeks than at one week (p<0.01). The BMD, bone microstructure parameters, MAR and bone strength were significantly decreased in the left tibias compared to the right tibias (all p<0.05). This simple and convenient local intraosseous siRNA injection model achieved gene silencing with very small quantities of siRNA over a short treatment period (≤7 days).

## Introduction

The bone microenvironment is very complex and includes not only osteocytes, osteoblasts, and osteoclasts but also bone marrow, mesenchymal stem cells, endothelial cells, adipocytes and nerves [1]. Bone can no longer be considered an isolated organ that is regulated independently of other homeostatic processes; on the contrary, bone represents an important metabolic organ that interfaces with adipose depots, skeletal muscle, pancreas, liver, testis, and the immune system through endocrine, paracrine and autocrine mechanisms [2,3]. The skeleton is an example of how a whole-organism approach to physiology can broaden the understanding of how a given organ functions and reveal connections to other organs [4]. An increasing number of studies have found that bone can secrete many biologically active substances, such as bone morphogenetic proteins, growth factors, adipokines, inflammatory cytokines, cardiovascular bioactive peptides, etc [1,5]. Through these bioactive substances, bone not only plays an important role in its own formation and remodeling [6,7] but also critically modulates systemic function [8].

There are several assays available to study the effects of molecules on bone cells in vitro [9,10]. However, because the complex interactions between cells are disrupted, these in vitro assays do not always reflect what occurs in vivo. A simple and rapid model is crucial to study the impact of one target gene, cytokine or signaling pathway on bone function.

In recent years, the development of transgenic technology has facilitated the study of bone functions [11]. However, the drawbacks of transgenic animal models are obvious, such as the long time requirement, high cost, substantial technical difficulties and high risk [12]. Additionally, the physical size of a transgenic mouse is small, which makes it difficult to study bone fracture healing or bone defect repair. A conditional knockout or tissue-specific gene knockout may be more complicated, especially in such a complex organ as the bone, which contains various cells. RNA interference (RNAi) allows for the experimental inhibition of gene expression, and thus the knockdown of proteins of interest, in mammalian cells in culture and in animals [13,14]. Harnessing RNAi will enable the exploration of the impact of a gene on pathophysiology, and this technique has great potential in the development of drugs designed to knock down the expression of any disease-causing gene, thereby greatly expanding the number of possible drug targets [15].

In the present study, we used insulin-like growth factor-1 (IGF-1) and recombinant human parathyroid hormone (1–34) (rhPTH (1–34)), two common bone anabolic agents that increase mesenchymal stem cell proliferation and osteoblast differentiation and result in enhanced bone formation primarily by acting through their cognate receptors, insulin-like growth factor-1 receptor (IGF-1R) and parathyroid hormone 1 receptor (PTH1R) [16,17]. We hypothesized that silencing these receptors via intraosseous injection of small interfering RNA (siRNA) would block the expression of the corresponding genes, thus influencing bone remodeling and decreasing BMD, MARs and mechanical stress. Therefore, in the current study, the rat left tibia received intraosseous injections of a *Pth1r* or *Igf-1r* siRNA transfection complex loaded in poloxamer 407 hydrogel, and the right tibia received the same volume of control siRNA. All the tibias received an intraosseous injection of rhPTH (1–34) or IGF-1 on the second day.

## Materials and Methods

### Animals and grouping

**Fig 1:**
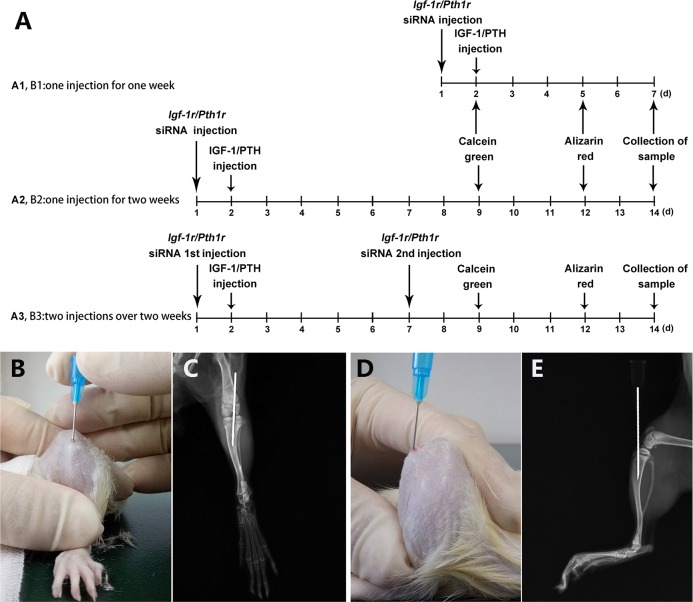
Animal Treatment Protocol and Images of the Intraosseous Injection Location. (A) Animal treatment protocol. (B) Anteroposterior and (D) lateral view of the minimally invasive puncture. The entry point was located at the front of the tibial intercondylar eminence, and the needle path penetrated the tibial plateau parallel to the long axis of the tibia. Representative (C) anteroposterior and (E) lateral X-ray radiographs of the tibia.

A total of forty-eight male Sprague-Dawley (SD) rats weighing 160 g each were divided into two groups; one group received *Pth1r* siRNA and rhPTH (n = 24), and the other group received *Igf-1r* siRNA and IGF-1 (n = 24). Then, according to the siRNA injection frequency and observation time, each group was divided as follows: 1) P-1-1 or I-1-1, one injection for one week; 2) P-1-2 or I-1-2, one injection for two weeks; and 3) P-2-2 or I-2-2, two injections over two weeks (Fig 1).

The rats were housed in a standard room with a 12-h light/dark cycle and given free access to food and water. All experimental procedures and protocols were approved by the Peking University Third Hospital Committee on Ethics for the Care and Use of Laboratory Animals.

### Preparation of injectable poloxamer 407 hydrogel loaded with rhPTH or IGF-1 and siRNA transfection complex

Poloxamer 407 (BASF, Ludwigshafen, Germany; 25% w/w) was added to isotonic phosphate-buffered saline (PBS, pH 7.4, 4°C) with gentle mixing until complete dilution [18]. Then, rhPTH (1–34) (Lilly, Fegersheim, France) or IGF-1 (ab198570, Abcam, Cambridge, USA) was added to the prepared poloxamer 407 solutions, and the final concentrations of IGF-1 and rhPTH (1–34) were 60 μg/ml and 80 μg/ml, respectively.

The *Pth1r* or *Igf-1r* siRNA (Sigma-Aldrich, USA) transfection complex was prepared according to the manufacturer’s instructions. In brief, 20 μg of siRNA (sequences are provided in Table 1) was dissolved in 20 μl of RNase-free water, followed by the addition of 10 μl of Entranster^TM^-in vivo (Engreen, Beijing, China) and gentle mixing; the mixture was then incubated for 15 min at room temperature. Finally, 20 μl of poloxamer 407 (BASF, Ludwigshafen, Germany, 50% w/w) was added to the solution with sufficient mixing.

**Table 1:** *Pth1r* and *Igf-1r* siRNA Sequences.

		forward (5'-3')	reverse (3'-5')
*Igf-1r*	NO. 1	GGAACUAUACGGCCCGGAUdTdT	AUCCGGGCCGUAUAGUUCCdTdT
	NO. 2	CAAGGAUAUUGGGCUUUAUdTdT	AUAAAGCCCAAUAUCCUUGdTdT
	NO. 3	GCAAUAACAUUGCCUCGGAdTdT	UCCGAGGCAAUGUUAUUGCdTdT
*Pth1r*	NO. 1	CGCUCUUUGGUGUCCACUAdTdT	UAGUGGACACCAAAGAGCGdTdT
	NO. 2	GGAGGUAUUUGACCGCCUAdTdT	UAGGCGGUCAAAUACCUCCdTdT
	NO. 3	GCAGUACCUUGUCCCGGAUdTdT	AAUCGGGACAAGGUACUGCdTdT
Negative Control		UUCUCCGAACGUGUCACGUTT	ACGUGACACGUUCGGAGAATT

### Local intraosseous injection of *Pth1r* or *Igf-1r* siRNA and rhPTH (1–34) or IGF-1

After 1 week of acclimation, all forty-eight SD rats were anesthetized by an intraperitoneal injection of 10% chloral hydrate (3.3 ml/kg). We located the entry point at the front of the tibial intercondylar eminence and inserted the needle parallel to the long axis of the tibia. A tibial radiograph ensured that the needle tip was in the medullary cavity (this validation step can be omitted after sufficient procedural skill is gained) (Fig 1).

On day 1, the left tibias received 20 μg of *Pth1r* or *Igf-1r* siRNA in 50 μl of transfection medium via intraosseous injection, while the right tibias received the same volume of control siRNA.

On day 2, all the rats received 100 μl of either rhPTH (1–34) or IGF-1 via an intraosseous injection in both the right and left tibias according to the experimental design.

On day 7, for the rats in groups P-2-2 and I-2-2, the left tibias received 20 μg of *Pth1r* or *Igf-1r* siRNA in 50 μl of transfection medium via intraosseous injection while the right tibias received the control siRNA transfection complex.

On day 7 for groups I-1-1 and P-1-1 and on day 14 for the other groups, the rats were euthanized with an overdose of anesthesia.

### Sequential fluorescence labeling

To ascertain the bone mineral apposition rates (MARs), double-fluorochrome labels were administered. Calcein green (Sigma, St. Louis, MO, USA; 8 mg/kg) and Alizarin red (Sigma, St. Louis, MO, USA; 20 mg/kg) were injected via the tail vein 6 days and 2 days before euthanasia, respectively.

### Bone mineral density (BMD) assessment

The BMD of the tibia was measured by dual-energy X-ray absorptiometry (DXA) using a small animal high-resolution collimator (Discovery™, Hologic Inc., Boston, MA, USA). The tibia was divided into five areas, and the region of interest (ROI) was set as the proximal two areas using a modified division method [19]. The same technician, who was blinded to the treatment assignments, conducted the DXA analyses.

### Micro-CT analysis

The tibias were placed in a sample holder with normal saline and scanned by micro-CT (Inveon, Siemens, Erlangen, Germany) at an effective pixel size of 8.82 μm, 80 kV/500 μA, and a 1500-ms exposure time in each of the 360 rotational steps. According to the guidelines set by the American Society for Bone and Mineral Research [20], the following parameters were calculated using an Inveon Research Workplace (Siemens): bone volume/total volume (BV/TV), trabecular thickness (Tb.Th), trabecular number (Tb.N) and trabecular separation (Tb.Sp) in the trabecular region (1–2 mm from the proximal epiphyseal plate).

### Biomechanical testing

To determine the mechanical properties of the tibia, a three-point bending test was performed on a mechanical testing system (Landmark, MTS Inc., Eden Prairie, MN, USA). Samples were thawed and continuously moistened with an isotonic saline solution. The two condyles of each tibia were placed in the free notches of an aluminum alloy base [21]. A preload of 1 N was used to fix the tibia on the device, and a bending force at a speed of 2 mm/min was applied for the test. The strength of the tibia was determined as the maximal force that caused complete failure of the tibia [22].

### Real-time PCR

IGF-1R and PTH1R expression levels were analyzed by real-time fluorogenic RT-PCR on a Roche LightCycler 2.0 (Germany). Three tibias randomly selected from each group were placed in liquid nitrogen and ground to a powder using a mixed-type ball mill (MM 400, Retsch GmbH, Retsch-Allee 1–5, 42781 Haan, Germany) for RNA extraction. A PrimeScript^TM^ RT Reagent Kit with gDNA Eraser (TaKaRa, Japan) was used for cDNA synthesis. The relevant cDNA was quantified with SYBR® Premix Ex Taq™ II (TaKaRa, Japan) upon amplification with specific primers (Invitrogen, USA; Table 2). The relative quantity of *Igf-1r* or *Pth1r* mRNA was normalized to GAPDH and calculated using the 2^–∆∆Ct^ method according to the manufacturer’s protocol.

**Table 2:** IGF-1R and PTH1R RT-PCR Primers.

	forward (5'-3')	reverse (3'-5')
IGF-1R	AAATACGGATCGCAAGTCGAG	TAGTTCCCTGGGTTTAGACGGT
PTH1R	TAAGCTTCGGGAGACCAATGC	AGCGGCACGAAGCACCAAC
GAPDH	CCTTCCGTGTTCCTACCCC	GCCCAGGATGCCCTTTAGTG

### Histology

Three specimens randomly selected from each group were fixed in 10% neutral buffered formalin, dehydrated in an increasing gradient of alcohol, and embedded in methylmethacrylate resin. Undecalcified sections of 30 μm were cut and ground parallel to the long axis of the tibia (EXAKT Cutting & Grinding System, Norderstedt, Germany). The MAR was calculated using fluorescence microscopy (DM3000, Leica Microsystems, Wetzlar, Germany) with Image-Pro 6.0 by measuring the mean distance between the 2 fluorescent labels divided by the time interval between injections [23]. After determination of the MAR, undecalcified sections were stained with Goldner’s trichrome and observed by light microscopy (E800, Nikon, Tokyo, Japan).

### Immunohistochemistry

Two tibias randomly selected from each group were fixed in 4% phosphate-buffered paraformaldehyde, decalcified in 10% ethylenediaminetetraacetic acid, and embedded in paraffin. The specimens were cut into 5-μm sections, which were then incubated with rat polyclonal anti-IGF-1R (1:100, ab39675, Abcam) or rat polyclonal anti-PTH1R antibodies (1:100, sc-20749, Santa Cruz Biotechnology) overnight at 4°C, followed by incubation with biotinylated anti-rat secondary antibody for 30 min. The sections were then treated with diaminobenzidine for microscopic visualization (E800, Nikon, Tokyo, Japan).

### Statistical analysis

All the data are presented as the mean ± standard deviation (SD) after statistical analysis with SPSS 16.0 (Chicago, IL, USA). The paired samples t-test was used to assess differences between the left and right tibias in each group. The independent samples t-test was used to compare PTH1R and IGF-1R gene expression between groups receiving one injection for one week and one injection for two weeks. A p value <0.05 was considered statistically significant.

## Results

The minimally invasive injections were successful in all the rats, and recovery was uneventful. No signs of infection or significant side effects or morbidity associated with the treatment were observed in any of the animals, and all the rats survived.

### Local intraosseous injection of *Pth1r* or *Igf-1r* siRNA silences PTH1R and IGF-1R

**Fig 2:**
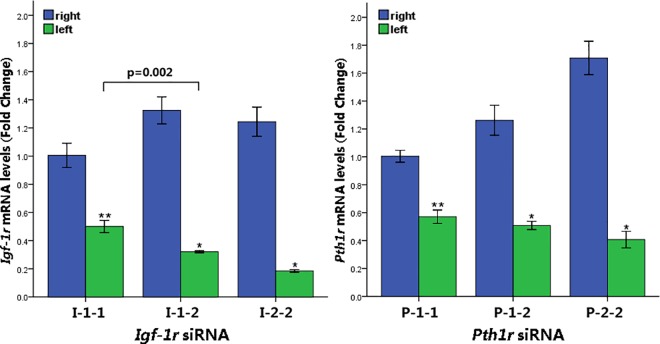
*Pth1r* and *Igf-1r* mRNA Expression Levels After siRNA Transfection. The real-time PCR results showed that *Pth1r* and *Igf-1r* mRNA expression levels were significantly diminished in the left tibia compared with the right tibia (I-1-1 and P-1-1, one injection for one week; I-1-2 and P-1-2, one injection for two weeks; I-2-2 and P-2-2, two injections over two weeks). *p<0.01, **p<0.05 vs. the right tibia.

The quantitative real-time PCR results indicated that local intraosseous injection of *Pth1r* or *Igf-1r* siRNA significantly diminished *Pth1r* or *Igf-1r* mRNA expression (Fig 2, p<0.05).

In the *Igf-1r* siRNA injection group, one injection for one week, one injection for two weeks and two injections over two weeks decreased *Igf-1r* mRNA expression by 49.85%, 75.72% and 85.06%, respectively. In the *Pth1r* siRNA injection group, one injection for a week, one injection for two weeks and two injections over two weeks decreased *Pth1r* mRNA expression by 46.28%, 59.69% and 76.01%, respectively.

Using poloxamer 407 hydrogel as a controlled release system prolonged the silencing effect of a single dose of siRNA; *Igf-1r* and *Pth1r* mRNA expression levels were lower at two weeks than at one week (p<0.01, p = 0.126, respectively).

### Local intraosseous injection of *Pth1r* or *Igf-1r* siRNA decreases PTH1R and IGF-1R expression

**Fig 3:**
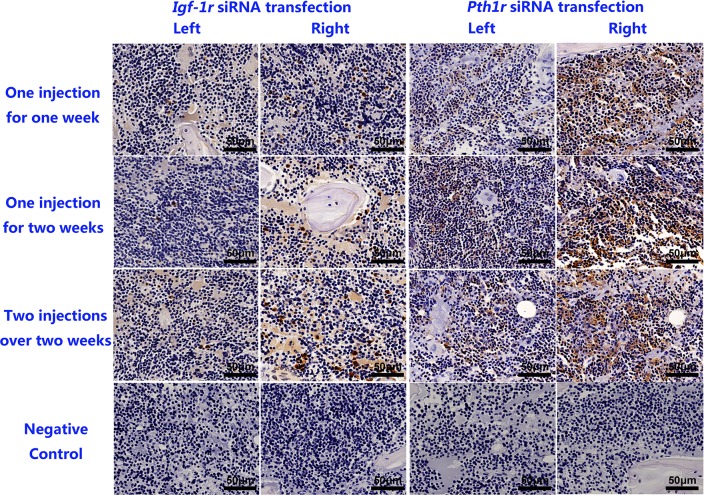
Immunohistochemical Staining for IGF-1R and PTH1R. The first three rows are representative images of the bone marrow of left tibias treated with *Igf-1r* siRNA and IGF-1 or *Pth1r* siRNA and rhPTH (1–34). The bottom row represents the isotype control for the anti-IGF-1R and anti-PTH1R antibodies. Scale bar = 50 μm.

As shown in Fig 3, PTH1R or IGF-1R expression levels were lower in the left tibia than in the right tibia, which did not receive an intraosseous injection of siRNA. There was no obvious difference between receiving one or two injections over a period of two weeks.

### Local intraosseous injection of *Pth1r* or *Igf-1r* siRNA decreases BMD

**Fig 4:**
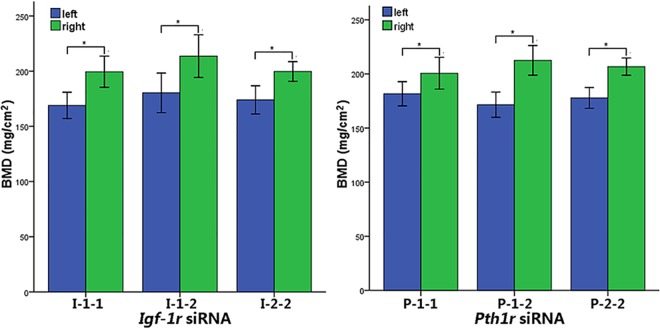
BMD Changes After Local Intraosseous Injection of *Pth1r* or *Igf-1r* siRNA. I-1-1 and P-1-1, one injection for one week; I-1-2 and P-1-2, one injection for two weeks; I-2-2 and P-2-2, two injections over two weeks. *p<0.01.

Quantification of BMD revealed that local intraosseous injection of *Pth1r* or *Igf-1r* siRNA significantly decreased BMD in the left tibia compared to the right tibia (Fig 4, p<0.05).

In the *Igf-1r* siRNA injection group, one injection for a week, one injection for two weeks and two injections over two weeks decreased the BMD by 15.26%, 15.58% and 12.89%, respectively. In the *Pth1r* siRNA injection group, one injection for a week, one injection for two weeks and two injections over two weeks decreased the BMD by 9.46%, 19.28% and 13.98%, respectively.

### Local intraosseous injection of *Pth1r* or *Igf-1r* siRNA attenuates bone microstructures

**Fig 5:**
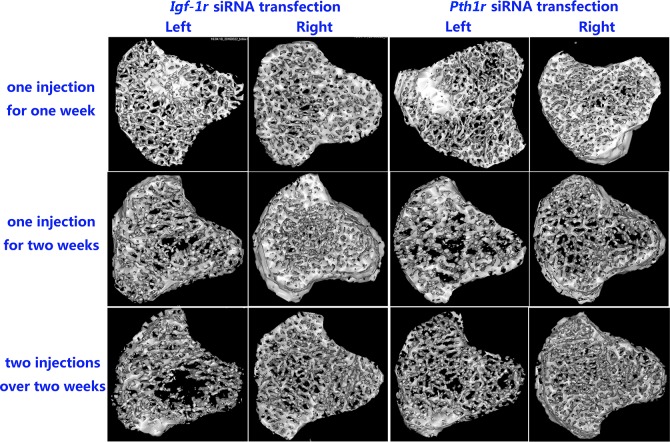
Representative Three-dimensional Trabecular Architecture of the Tibia After *Pth1r* or *Igf-1r* siRNA Injection. In the *Igf-1r* siRNA transfection groups, the trabeculae in the left tibias treated with *Igf-1r* siRNA and IGF-1 were dramatically weakened compared to those in the right tibias treated with IGF-1 only. In the *Pth1r* siRNA transfection groups, the trabeculae in the left tibias treated with *Pth1r* siRNA and rhPTH (1–34) were also significantly weakened compared to those in the right tibias treated with rhPTH (1–34) only.

Micro-CT imaging showed that local intraosseous injection of *Pth1r* or *Igf-1r* siRNA decreased the bone volume and attenuated the microstructure of the left tibia compared to the right tibia (Fig 5), which was further verified by quantitative analysis of the micro-CT results (Table 3, p<0.05).

**Table 3:** Quantitative μCT Analysis of Tibial Microstructural Parameters.

			*Igf-1r* siRNA			*Pth1r* siRNA	
			Left	Right		Left	Right
BV/TV (%)		**I-1-1**	35.12±3.41*	52.67±5.59	**P-1-1**	41.99±5.24*	58.62±4.65
		**I-1-2**	37.11±3.22**	57.59±7.41	**P-1-2**	36.40±5.49*	53.16±2.38
		**I-2-2**	32.07±6.65*	46.75±3.37	**P-2-2**	33.36±5.52**	52.90±5.54
Tb.Th (μm)		**I-1-1**	56.20±4.34*	73.72±7.06	**P-1-1**	59.19±3.02*	83.72±3.68
		**I-1-2**	60.36±3.24*	84.94±8.86	**P-1-2**	56.62±2.72**	88.83±2.21
		**I-2-2**	57.01±3.63*	67.57±4.55	**P-2-2**	55.86±3.65**	90.32±5.19
Tb.N (1/mm)		**I-1-1**	5.35±0.42**	7.11±0.15	**P-1-1**	5.42±0.36*	6.91±0.30
		**I-1-2**	6.00±0.29*	7.25±0.28	**P-1-2**	5.78±0.55**	7.62±0.26
		**I-2-2**	4.99±0.29**	6.91±0.79	**P-2-2**	5.03±0.41**	6.88±0.29
Tb.Sp (mm)	**I-1-1**		0.99±0.10**	0.57±0.09	**P-1-1**	0.82±0.08*	0.59±0.05
	**I-1-2**		0.91±0.06*	0.56±0.09	**P-1-2**	1.11±0.21**	0.67±0.04
	**I-2-2**		1.28±0.31**	0.69±0.05	**P-2-2**	1.24±0.28**	0.74±0.02

Data are presented as the mean±SD, n = 8 in each group.

*p<0.05

**p<0.01 vs. the right tibia. BV/TV: bone volume/tissue volume; Tb.Th: average trabecular thickness; Tb.N: trabecular number; Tb.Sp: average trabecular separation.

### Local intraosseous injection of *Pth1r* or *Igf-1r* siRNA reduces bone strength

**Fig 6:**
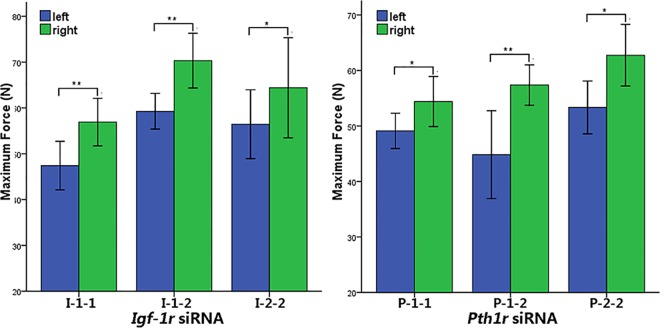
Maximum Force in the Three-point Bending Biomechanical Test. In the *Igf-1r* and *Pth1r* siRNA transfection groups, the mechanical properties of the left tibias, which received *Igf-1r* or *Pth1r* siRNA injections, were dramatically diminished compared to the right tibias (I-1-1 and P-1-1, one injection for one week; I-1-2 and P-1-2, one injection for two weeks; I-2-2 and P-2-2, two injections over two weeks). *p<0.05 and **p<0.01.

Local intraosseous injection of *Pth1r* or *Igf-1r* siRNA significantly reduced the maximum load force of the left tibias compared to the right tibias (Fig 6, p<0.05).

In the *Igf-1r* siRNA injection group, one injection for one week, one injection for two weeks and two injections over two weeks decreased the maximum force by 16.68%, 15.71% and 12.33%, respectively. In the *Pth1r* siRNA injection group, one injection for one week, one injection for two weeks and two injections over two weeks decreased the maximum force by 9.73%, 21.86% and 11.81%, respectively.

### Local intraosseous injection of *Pth1r* or *Igf-1r* siRNA influences bone formation

**Fig 7:**
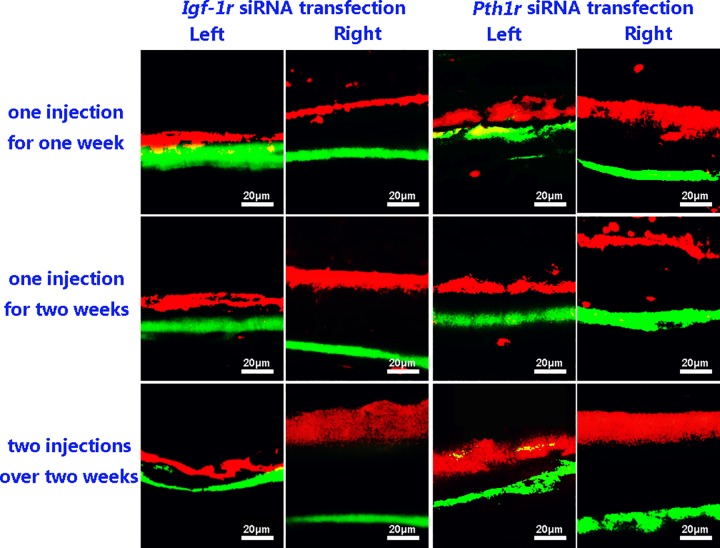
Representative Images of Double Fluorochrome Labeling. In the *Igf-1r* siRNA transfection group, the distance between the two labels in the left tibias treated with *Igf-1r* siRNA and IGF-1 was significantly shorter than that in the right tibias treated with IGF-1 only. In the *Pth1r* siRNA transfection group, this distance was also markedly decreased in the left tibias treated with *Pth1r* siRNA and rhPTH (1–34) compared to the right tibias treated with rhPTH (1–34) only. Scale bar = 20 μm.

**Fig 8:**
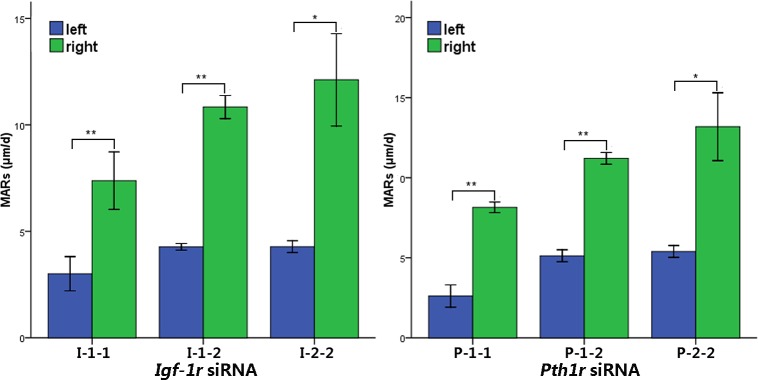
Quantification of MARs. In the *Igf-1r* and *Pth1r* siRNA transfection groups, quantification of the MAR revealed that *Igf-1r* or *Pth1r* siRNA injection in the left tibias dramatically reduced the bone formation rate compared to the right tibias treated with IGF-1 or rhPTH (1–34) only (I-1-1 and P-1-1, one injection for one week; I-1-2 and P-1-2, one injection for two weeks; I-2-2 and P-2-2, two injections over two weeks). *p<0.05 and **p<0.01.

Fluorescent labeling indicated that local intraosseous injection of *Pth1r* or *Igf-1r* siRNA decreased bone formation in the left tibia compared to the right tibia (Fig 7), which was further verified by the MAR (Fig 8, p<0.01).

In the *Igf-1r* siRNA injection group, one injection for one week, one injection for two weeks and two injections over two weeks significantly decreased the MAR by 59.35%, 60.61% and 64.68%, respectively. In the *Pth1r* siRNA injection group, one injection for one week, one injection for two weeks and two injections over two weeks decreased the MAR by 67.95%, 54.28% and 59.11%, respectively.

**Fig 9:**
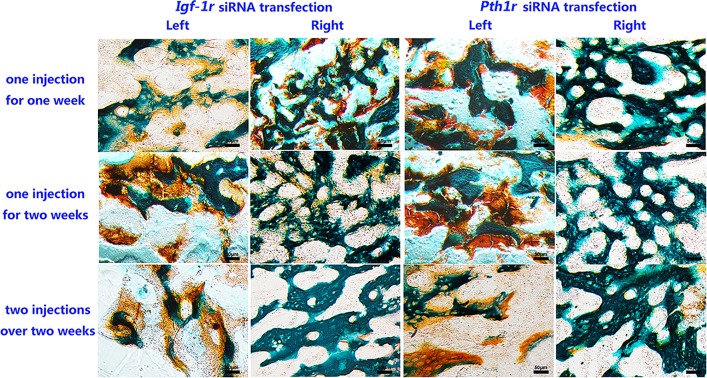
Representative Photomicrographs of Undecalcified Sections Stained with Goldner’s Trichrome. In the left tibias treated with *Igf-1r* or *Pth1r* siRNA, osteoid accounted for a very large proportion, which was obviously greater than that in the right tibias, in which the majority was mature trabecular bone. Mature trabecular bone and osteoid are stained green and red, respectively. Scale bar = 50 μm.

Using Goldner’s trichrome staining, mature trabecular bone and the osteoid were stained green and red, respectively [24], which showed that local intraosseous injection of *Pth1r* or *Igf-1r* siRNA significantly decreased mature trabecular bone formation in the left tibia compared to the right tibia (Fig 9).

## Discussion

The bone microenvironment is very complex and includes multiple cell types that reciprocally interact with each other via paracrine signaling or cell-to-cell communication with direct contact [1,10]. It is difficult to mimic this complex environment in vitro. The calvarial injection method, originally described by Boyce et al. [25] is valuable for studying the effects of substances on bone metabolism in vivo [26,27]. Li et al. reported that a small dose of PTH or PGE2 injected daily for 10 days via a small needle passing through a steel cannula inserted into the marrow cavity of the proximal tibia promotes bone formation [28]. This local injection model combines the numerous advantages of in vivo models (systemic injection) and in vitro models for assessing agents with anabolic skeletal activity [28]. In our previous study, we confirmed that a single dose of simvastatin via intraosseous injection promotes bone formation [29,30].

In this study, we developed a rapid and minimally invasive method for the local intraosseous injection of siRNA to assess the effects on bone. This proof-of-principle study showed that a single intraosseous injection of *Pth1r* or *Igf-1r* siRNA significantly diminished *Pth1r* or *Igf-1r* mRNA and protein expression levels. The decreased BMD, diminished bone formation, and changes to the local bone microstructure and biomechanical properties further verified the silencing efficacy of this procedure.

The ability of siRNA to silence specific target genes not only offers a tool to study gene function but also represents a novel approach for the treatment of disease [31]. Many studies have used siRNA technology to explore the mechanism or treatment of diseases such as autoimmune diseases, viral infections, cancer, and dominant genetic disorders [32]. Compared with systemic delivery methods, the local delivery of siRNA/shRNA has many advantages, such as targeting specific tissues or organs, mimicking a conditional gene knockout effect, or enabling the development of certain disease models. The local delivery of siRNA is ideal for diseases in which the target sites are easily accessible. In this model, very small quantities of siRNA were delivered locally into bone, thus generating a high local concentration that was sufficient to produce biological effects in a short period of time.

The siRNA can be delivered locally in a naked form, with chemical modifications, or in formulations with viral or non-viral vectors, such as liposomes and nanoparticles [33]. During the in vivo delivery process, siRNA encounters substantial obstacles while attempting to reach the target cells. For example, ribonucleases may limit the half-life of naked siRNA molecules in the circulation to less than 1 min [34]. The siRNA vectors (Entranster^TM^-in vivo) used in this study were commercial dendrimer nanoparticles containing many amino groups that protonate at physiological pH. The protonated amino groups can then neutralize the electric charge on the surface of the siRNA, allowing the siRNA molecules to be compacted into relatively smaller structures to prevent nuclease degradation. The nanometer-scale transfection reagent has unique characteristics, such as providing stronger siRNA protection and lower cytotoxicity [34].

The intraosseous injection route is an alternative to the intravenous route; agents injected via the intraosseous route reach the circulation rapidly, similarly to agents injected via the intravenous route [35–37]. Poloxamer 407 is biocompatible and stable and displays low toxicity and weak immunogenicity, making it suitable as a vehicle for a controlled drug delivery system. Poloxamer 407 also demonstrates reversible thermosensitive gelation (sol-gel characteristics), which is convenient for handling and provides ease of application [38].

In this proof-of-principle study, we used IGF-1 and rhPTH (1–34), two common bone anabolic agents that increase mesenchymal stem cell proliferation and osteoblast differentiation and result in enhanced bone formation primarily through their cognate receptors, IGF-1R and PTH1R [16,17]. In the current study, we developed a unique delivery approach by which siRNA and dendrimer nanoparticles could be delivered directly and locally to the target tissue. Our results suggested that we can obtain sufficient gene silencing with this simple and convenient method.

According to the results of the current study, a one-time intraosseous injection of siRNA achieved a silencing effect at one week, and this effect was sustained for at least for two weeks. Compared to one injection for one week, the silencing efficacy of one injection for two weeks was much better, possibly due to the use of poloxamer 407 hydrogel, which facilitated the sustained release of the siRNA transfection complex. Although the silencing efficacy of two injections over two weeks was significantly higher than that of one injection for two weeks, the skeletal biological properties, such as the BMD, microstructure and strength, did not change significantly. To obtain improved gene silencing results, an injection frequency of once per week may be better and may be adjusted according to the length of the study period.

Considering the complex composition of cells in the bone and that the main constituent of bone is mineral, there is a relatively low quantity of mRNA and protein. We did not detect related downstream genes after siRNA silencing; we only observed the biological effects induced by local siRNA silencing. This is a limitation of our study. We will detect changes in related downstream genes in a future study using this model.

There are some advantages of this model. Compared to conventional in vivo systemic injection models, this model is convenient and rapid and uses less siRNA, allowing for a high effective local dose of siRNA in the bone and avoiding possible negative side effects in other tissues. Using this model, we can rapidly screen new molecules that may play an important role in the modulation of bone metabolism. Furthermore, the contralateral tibia can be used as an internal control in the same animal.

## Conclusions

This simple and convenient local intraosseous siRNA injection model has the numerous advantages of in vivo models for assessing the influence of genes on skeletal activities and is capable of providing strong gene silencing with small quantities of siRNA over a short treatment period.

## Abbreviations

siRNA: small interfering RNA
BMD: bone mineral density
MARs: mineral apposition rates
RNAi: RNA interference
SD rats: Sprague-Dawley rats
DXA: dual-energy X-ray absorptiometry
ROI: region of interest
BV/TV: bone volume/total volume
Tb.Th: trabecular thickness
Tb.N: trabecular number
Tb.Sp: trabecular separation
IGF-1: insulin-like growth factor-1
IGF-1R: insulin-like growth factor-1 receptor
rhPTH (1–34): recombinant human parathyroid hormone (1–34)
PTH1R: parathyroid hormone 1 receptor.
